# Renal anemia and hydration status in non-dialysis chronic kidney disease: Is there a link?

**DOI:** 10.25122/jml-2019-0002

**Published:** 2018

**Authors:** Simona Hildegard Stancu, Ana Stanciu, Mariana Lipan, Cristina Capusa

**Affiliations:** 1. “Carol Davila” University of Medicine and Pharmacy, Nephrology Dept., Bucharest, Romania; 2. “Dr. Carol Davila” Teaching Hospital of Nephrology, Bucharest, Romania

**Keywords:** Chronic Kidney Disease, Fluid overload, Hemoglobin, Iron deficiency, Renal anemia, BCM: Body composition monitor, CKD: Chronic kidney disease, CRP: C-reactive protein, CVD: Cardiovascular diseases, (e)GFR: (Estimated) Glomerular filtration rate, Epo: Erythropoietin(s), ESA: Erythropoiesis stimulating agents, ESKD: End-stage kidney disease, Hemoglobin: Hb, OH: Overhydration, TSAT: Transferrin saturation

## Abstract

**Rationale:** Anemia, a common feature in chronic kidney disease (CKD), has multiple contributors to its pathogenesis. Besides the well recognized erythropoietin and iron deficiencies, hydration status might be involved.

**Objective:** To assess the prevalence and correlations of anemia, iron deficiency and overhydration in patients with stage 2 to 5 CKD.

**Methods and Results:** This cross-sectional study enrolled 125 erythropoietin and iron therapy naïve non-dialysis CKD patients, without a identifiable cause of anemia.

Parameters of hematological, iron, inflammatory and nutritional status were measured. The overhydration parameter (OH) assessed by bioimpedance spectroscopy was used to characterize hydration status.

The prevalence of decreased hemoglobin (Hb) <110g/L increased along CKD stages from 0% to 40% (*p*=0.008). Fluid overload (OH >1L) and lower serum albumin (<40g/L) were more common in stage 5 versus stage 3 CKD (53% vs. 10%, *p*<0.001, and 27% vs. 3%, *p*=0.02, respectively), suggesting a potential dilutional reduction in serum proteins. Conversely, decreased iron stores (ferritin <100mcg/L) and iron availability (transferrin saturation, TSAT<0.20) were similarly prevalent irrespective of kidney function decline.

Hemoglobin was positively correlated with estimated glomerular filtration rate (eGFR), serum albumin, and transferrin saturation, but inversely with OH. However, in a model of multiple linear regression which explained 32% of hemoglobin variation, only eGFR and overhydration remained the independent predictors of anemia.

**Discussion:** As fluid overload is a common denominator for hemoglobin and TSAT levels, and is closely related to the declining kidney function, it should be considered in the management of renal anemia, at least in advanced CKD.

## Introduction

Anemia is common in the course of chronic kidney disease (CKD) and end-stage kidney disease (ESKD), due to either disturbances induced by the glomerular filtration rate (GFR) decline (as erythropoietin deficiency, iron metabolism impairment, inflammation, uremic toxins retention, vitamin deficiencies, secondary hyperparathyroidism, reduced red cells survival) or the underlining disease or various chronic therapies [1]. Decreased renal production of erythropoietin (Epo) is its main mechanism, but increased iron losses and impaired absorption of dietary iron are also present. In addition to true iron deficiency, reduced iron availability characterized by altered iron release from body stores that is inadequate for erythropoiesis, mainly due to inflammation-induced reticulo-endothelial cell iron blockade, also contributes [2,3]. CKD patients are 25% more likely to develop anemia than their non-CKD counterparts [4]. Anemia occurs early in the development of kidney disease and worsens with declining kidney function, as rising prevalence is found at each stage of the disease [4,5,6]. Moreover, higher risk for cardiovascular disease, mortality, and decreased quality of life was associated with renal anemia [4,6].

Consequently, erythropoiesis stimulating agents (ESA) and iron are widely prescribed to correct anemia in CKD, but both approaches were related to unwanted side effects [7]. Large interventional trials with recombinant human Epo, targeting near-normal hemoglobin levels, did not find any benefit on survival [1], while the risk for stroke, thromboembolic events and recurring cancers was higher [8]. On the other hand, intravenous iron (increasingly used in the last years) could lead to long-term complications related to triggering oxidative stress or tissue deposition, for example [8].

Therefore, an accurate diagnosis of anemia is essential to guide the appropriate treatment and to avoid unnecessary medications with potential to induce harm. Unfortunately, the criteria to define anemia and iron status in CKD are less clear. While the *National Kidney Foundation *defines anemia as hemoglobin (Hb) <135g/L (13.5g/ dL) in men and <120g/L (12g/dL) in women [6], a recent updated guideline of the United Kingdom *Renal Association *recommends the assessment for causes and weighing therapeutic options when Hb decreases below 110g/L (11g/dL) [9]. Moreover, the optimal target hemoglobin levels for patients with various stages of CKD are even more doubtful [10].

Among the complications related to reduced GFR, fluid overload is also common due to impairment in the kidney ability to handle water and salt, with subsequent water retention. Chronic fluid overload was found even in early stages of CKD and may significantly contribute to arterial hypertension, accelerated arteriosclerosis and the high prevalence of left ventricular hypertrophy and congestive heart failure [11,12].

Since anemia diagnosis and management rely on levels of serum variables like Hb, transferrin and ferritin, all of which are dependent on the volemia, we thought to evaluate the relationships between these parameters and the hydration status in non-dialysis CKD patients.

## Methods

### Subjects

In this observational, cross-sectional study performed in a specialized, tertiary-care center of Nephrology, 125 in-patient adults with various stages of non-dialysis CKD (8% stage 2, 47% stage 3, 33% stage 4, and 12% stage 5 CKD) were prospectively enrolled over a period of three months. All patients provided written informed consent prior to any study procedures. The research fully complied with the Good Clinical Practice guidelines and the Declaration of Helsinki adopted in 1964, with its following revisions.

CKD was diagnosed if urinary albumin-to-creatinine ratio >30mg/g and/or eGFR <1mL/s (60mL/min) persisted over three months. Exclusion criteria were: previous treatments with Epo and iron, anemia of identifiable cause (hemolytic, megaloblastic, recent bleedings), active infections and inflammatory states, active neoplasia, severe liver diseases (elevated serum transaminases over 3-fold the upper limit of the laboratory range), immunosuppressive treatments, nephrotic syndrome, and congestive heart failure.

### Laboratory assessments

Fasting blood samples were drawn and the routine parameters were analyzed with commercially available kits on multiparameter analyzers. Blood hemoglobin concentration was used as indicator for anemia. Iron status was assessed by measurement of serum ferritin (iron store) and transferrin saturation (TSAT - adequacy of iron for erythropoiesis). C-reactive protein (CRP), as marker of the inflammation, and serum albumin, as marker of nutritional status, were also measured. Estimated GFR was calculated based on serum creatinine by the abbreviated MDRD equation.

Bioimpedance spectroscopy with a portable body composition monitor device (BCM Fresenius®) was used to assess the hydration status. Overhydration parameter (OH) provided by BCM describes the fluid overload located almost exclusively in the extracellular space and is consider to characterize hydration status (normal range: −1.1 to +1.1 liter, according to the 10th and 90th percentile of the measurements in a cohort of healthy subjects) [13].

### Statistical analysis

Data were expressed as percentages, mean ± standard deviation (SD) or median with 95%CI (according to the normal or non-normal distribution, respectively), and compared using Chi2, t Student or Mann-Whitney tests, as appropriate. Spearman correlation test and multiple linear regression (with logarithmic transformed variables for the skewed data) were performed to assess correlations. StatistiXL version 1.x for EXCEL and GraphPad Prism version 3 were used.

A *p* value < 0.05 was considered significant.

## Results

General characteristics of the subjects are presented in Table 1.

**Table 1:** General characteristics of the subjects (N=125)

Parameter	
Age (years)†	60 (56 to 61)
Gender (% males)	60%
Diabetes mellitus (%)	26%
Cardiovascular diseases (%)	50%
CKD vintage (years)†	3 (3.8 to 5.8)
Primary kidney disease	
Vascular nephropathies (%)	34%
Glomerulopathies (%)	30%
Tubulo-interstitial nephropaties (%)	26%
Hereditary nephropathies (%)	10%
eGFR (mL/s)†	0.52 (0.50 to 0.60)
Body mass index (kg/m^2^)†	26.6 (26.2 to 28.2)
Body mass index >25kg/m^2^ (%)	66%
Hemoglobin (g/L)*	12.5 ± 2.0
Serum ferritin (mcg/L)†	154 (174 to 228)
Transferrin saturation*	22 ± 8
Optimal iron status‡ (%)	32%
Serum albumin (g/L)*	44 ± 0.3
Serum albumin <40g/L (%)	11%
C-reactive protein (nmol/L)†	28.6 (41.9 to 70.5)
Overhydration (L)†	−0.2 (−0.3 to 0.4)
Fluid overload (% OH>1L)	22%

* : mean ± standard deviation;

† : median (95% confidence interval);

‡ : defined by serum ferritin 100-500mcg/L and transferrin saturation >0.20. CKD: chronic kidney disease; eGFR: estimated glomerular filtration rate; OH: overhydration parameter.

**Figure 1:**
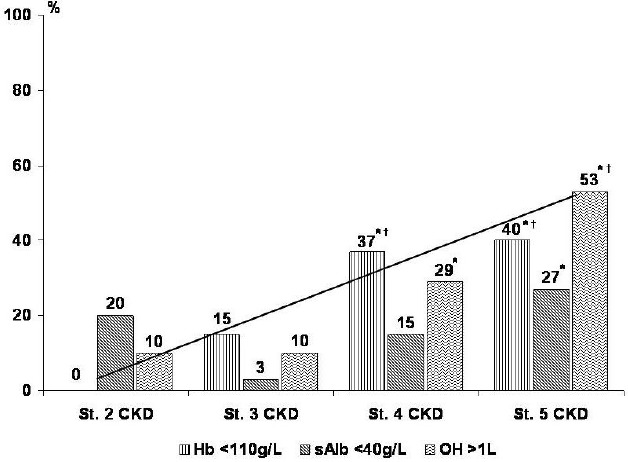
Prevalences of anemia, fluid overload and lower serum albumin stratified by the stage of chronic kidney disease. (St.: stage; CKD: ckronic kidney disease; Hb: hemoglobin; sAlb: serum albumin; OH: overhydration parameter; *: *p*<0.05 versus stage 3 CKD; †: *p*<0.05 versus stage 2 CKD)

The overall prevalence of anemia, defined as Hb <110g/L (<11g/dL) in accordance with the Renal Association updated guideline [9], was 24%, but it increased along CKD stages (*p*=0.008). Higher percentages of overhydration (OH >1L) and lower serum albumin levels [<40g/L (<4g/dL)] were also seen in advanced CKD: more subjects in stage 5 CKD had fluid overload than those in stage 3 and 2 CKD (*p*<0.001, and *p*=0.01, respectively), and lower serum albumin versus stage 3 CKD (*p*=0.02). A higher prevalence of overhydration was found even in stage 4 as compared to stage 3 CKD (*p*=0.01) (Figure 1).

In the whole group, the prevalence of decreased iron stores [ferritin <100mcg/L (100ng/mL)] and iron availability for erythropoiesis [ferritin >100mcg/L (100ng/mL) and TSAT <0.20 (20%)] were 32% and 33%, respectively, without differences across CKD stages.

Subjects with anemia had lower eGFR and TSAT, but higher OH and more prevalent diabetes mellitus (Table 2).

**Table 2:** Comparisons between subjects with and without anemia

Parameter	Subjects with Hb<110g/L (n=30)	Subjects with Hb>110g/L (n=95)	p
Age (years)†	62 (51 to 65)	60 (55 to 62)	0.94
Gender (% males)	50%	62%	0.24
CKD vintage (years)†	4 (3.3 to 5.8)	3 (3.7 to 6.1)	0.46
**Diabetes mellitus (%)**	**40%**	**21%**	**0.04**
CVD (%)	60%	45%	0.16
BMI (kg/m^2^)†	26.6 (25.0 to 29.7)	26.6 (26.0 to 28.2)	0.86
**eGFR (mL/s)†**	**0.33 (0.32 to 0.47)**	**0.56 (0.54 to 0.66)**	**<0.001**
Hemoglobin (g/L)*	101 ± 8	133 ± 16	<0.001
Serum ferritin (mcg/L)†	173 (132 to 233)	150 (175 to 239)	0.62
TSAT*	**0.19 ± 0.08**	**0.23 ± 0.08**	**0.02**
Serum albumin (g/L)*	45 ± 4	44 ± 3	0.27
CRP (nmol/L)†	28.6 (31.4 to 65.7)	28.6 (40.9 to 76.2)	0.80
**Overhydration (L)†**	**0.7 (0.2 to 2.0)**	**−0.3 (−0.6 to 0.1)**	**0.002**
**OH>1L (%)**	**43%**	**16%**	**0.002**

* : mean ± standard deviation;

† : median (95% confidence interval).

Hb: hemoglobin; CKD: chronic kidney disease; CVD: cardiovascular diseases; BMI: body mass index; eGFR: estimated glomerular filtration rate; TSAT: transferrin saturation; CRP: C-reactive protein; OH: overhydration parameter.

In addition, when divided according to the hydration status, more patients with hypervolemia (OH>1L, n=28) had anemia as compared to both euvolemic (OH between −1L and +1L, n=65) and hypovolemic (OH<−1L, n=32) subgroups. The prevalence of anemia in patients with higher OH was five times as much as in hypovolemic (46% vs. 9%, *p*<0.001) and double than in euvolemic (46% vs. 22%, *p*=0.02) subjects.

**Figure 2:**
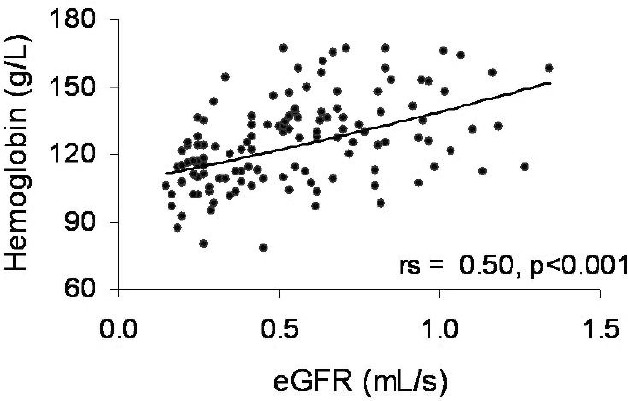
The positive correlation between serum hemoglobin and kidney function. (eGFR: estimated glomerular filtration rate; rs: Spearman’s rank correlation coefficient)

**Figure 3:**
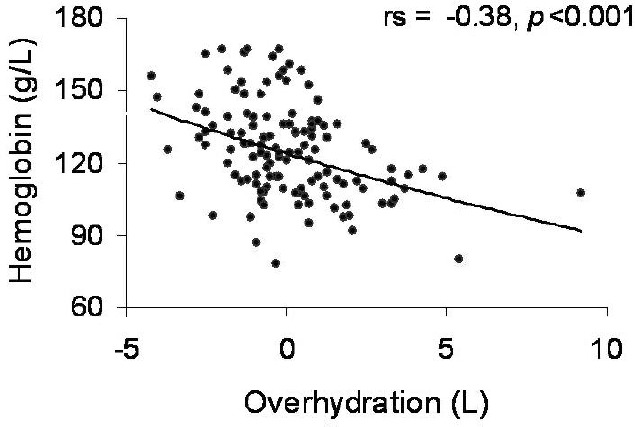
The inverse correlation between serum hemoglobin and hydration status. (OH: overhydration parameter at bioimpedance spectroscopy; rs: Spearman’s rank correlation coefficient)

In univariate analysis, Hb correlated directly with eGFR (Figure 2), TSAT (rs=0.24, *p*=0.008) and serum albumin (rs=0.28, *p*=0.001), but inversely with overhydration (Figure 3). Instead, no correlations with age, CKD vintage, serum ferritin, and the investigated inflammation marker (CRP) were found.

In a model of multiple linear regression which explained 32% of hemoglobin variation (build from the parameters which showed significant diferrences at previous analyses), only overhydration and eGFR were retained as the independent predictors of anemia (Table 3).

**Table 3:** The predictors of anemia

Predictor	Std. Beta	Coefficient	95%CI for coefficient	p
**(Constant)**		7.39	5.52 to 9.25	<0.001
**Ln(eGFR)**	0.43	1.53	0.99 to 2.08	<0.001
**OH**	−0.29	−0.29	−0.44 to −0.13	<0.001

N=125; Adjusted R^2^: 0.32; *p*<0.001

Dependent variable: serum hemoglobin

Variables entered the first step (backward stepwise): Ln(eGFR), serum albumin, transferrin saturation, and overhydration

Std. Beta: standardized beta; Ln(eGFR): natural logarithm of estimated glomerular filtration rate; OH: overhydration parameter

## Discussion

Chronic kidney disease is a growing public health problem since it affects in average one out of ten persons worldwide. A recent epidemiologic study in our country reported an overall CKD prevalence of almost 7% [14], so one can estimate that around 1.3 millions Romanian has a degree of kidney impairment. In conjunction with the improved life expectancy of patients with CKD, due to more efficient management of the main underlying causes (i.e. diabetes mellitus, arterial hypertension, and cardiovascular diseases) as well as to the better availability of renal replacement therapy for kidney failure, the consequence will be to encounter more an more cases with decreased GFR in the daily medical practice. Therefore, one should expect a rising number of complications related to the declining kidney function, including renal anemia and fluid overload, which will need to be properly addressed. These reasons support the importance of current research, especially since, to the best of our knowledge, it is the first study in Romania that directly analyzed the correlations of serum hemoglobin and iron metabolism parameters with fluid overload in patients with CKD who do not yet require dialysis.

As presumed based on the pathophysiology of renal anemia and in accordance with data from the literature [15,16], high prevalence of anemia was seen in our patients starting from stage 3 CKD and gradually increasing with falling eGFR. Even if is theoretically conceivable to attribute this pattern to the Epo deficit, it worth to note the presence of iron status abnormalities in almost two third of the studied group, which highlights the role of other mechanisms as well. Iron deficiency as a major contributor to renal anemia was previously sustained [17,18], and the present data are concordant.

However, the novel and most interesting finding in this study is the trend of volume overload prevalence which runs almost parallel that of the anemia along the CKD stages. The seriousness of hyperhydration is from long-time well known in ESKD patients [19], but more recently was also discussed in non-dialysis CKD. For example, Hung S-C et al. found euvolemia in a little less than half (48%) from a cohort of 338 adults with stage 3 to 5 non-dialysis CKD [20], percentage which is very similar to our observations (52% patients with OH in normal range). In the same study, significant correlations of overhydration with markers of nutritional and inflammatory status (lower serum albumin and higher interleukin-6 and tumor necrosis factor-α) were reported in addition to those with systolic blood pressure, cardiovascular diseases, and diabetes mellitus. Contrary to the present study findings, the relationship with GFR was minor and not retained after multivariate analysis, while a strong positive association with proteinuria was more clear [20]. We did not test proteinuria, but on a theoretic ground, the relation of water retention with both proteinuria and glomerular filtration seems straightforward.

The fact that as higher the hydration status parameter is the hemoglobin is lower, points to the need of make the distinction between the true anemia and the influence of hemodilution even in CKD patients without overt signs of volume overload.

Despite the strength resulted from the inclusion of subjects never before treated with ESA and intravenous iron, the current study has limitations. Firstly, the cross-sectional design does not allow any causality speculations, and the small sample size and single center character hamper the possibility of generalizability. Another drawback is the lack of data about the dietary salt intake and diuretic treatment. However, the association between anemia and fluid overload can be argued, and this warrants further studies.

In conclusion, as fluid overload is a common denominator for serum hemoglobin and transferrin saturation, and is closely related to the reduction in kidney function, it is an important factor to consider in the management of renal anemia, at least in advanced chronic kidney disease. In other words, it is advisable for clinicians to carefully assess hydration status during the diagnosis work-up for renal anemia whenever the GFR is below 0.50mL/s (30mL/min) and to decide the prescription of antianemic medications only after correction of the “dilutional” component of the anemia.

## Conflict of interest

The authors confirm that there are no conflicts of interest.
